# Full-Inorganic Flexible Ag_2_S Memristor with Interface Resistance–Switching for Energy-Efficient Computing

**DOI:** 10.1021/acsami.2c11183

**Published:** 2022-09-14

**Authors:** Yuan Zhu, Jia-sheng Liang, Xun Shi, Zhen Zhang

**Affiliations:** † Division of Solid-State Electronics, Department of Electrical Engineering, Uppsala University, Uppsala 75121, Sweden; ‡ State Key Laboratory of High Performance Ceramics and Superfine Microstructure, Shanghai Institute of Ceramics, Chinese Academy of Sciences, Shanghai 200050, China

**Keywords:** flexible memristor, Ag_2_S, interface resistance−switching, switching energy, energy-efficient computing

## Abstract

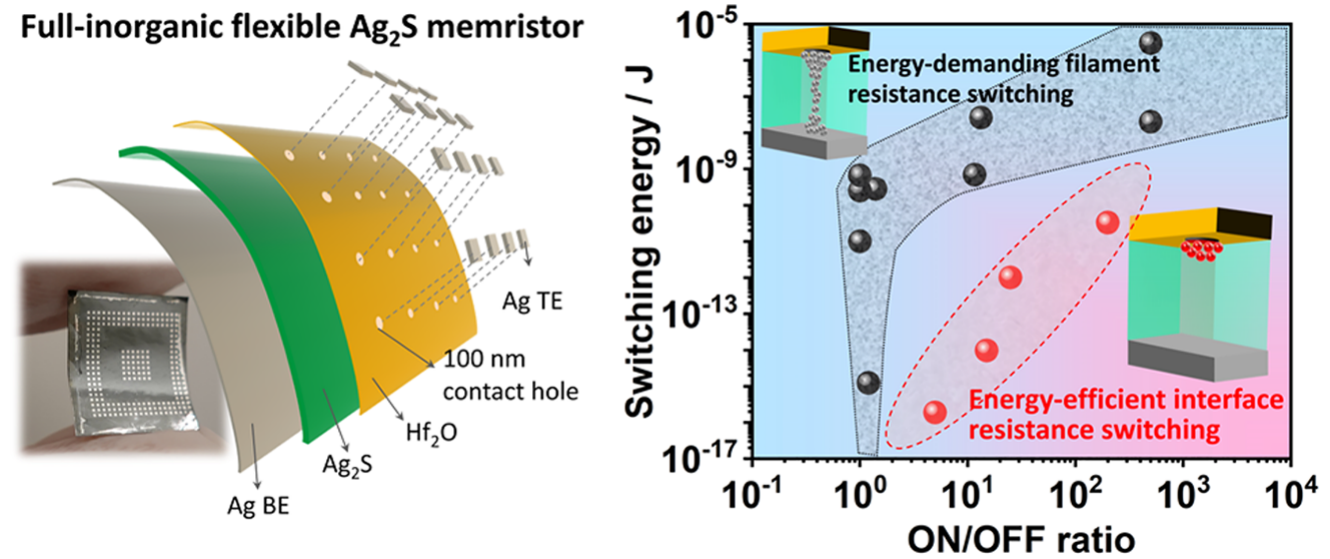

Flexible memristor-based neural network hardware is capable of implementing parallel computation within the memory units, thus holding great promise for fast and energy-efficient neuromorphic computing in flexible electronics. However, the current flexible memristor (FM) is mostly operated with a filamentary mechanism, which demands large energy consumption in both setting and computing. Herein, we report an Ag_2_S-based FM working with distinct interface resistance–switching (RS) mechanism. In direct contrast to conventional filamentary memristors, RS in this Ag_2_S device is facilitated by the space charge-induced Schottky barrier modification at the Ag/Ag_2_S interface, which can be achieved with the setting voltage below the threshold voltage required for filament formation. The memristor based on interface RS exhibits 10^5^ endurance cycles and 10^4^ s retention under bending condition, and multiple level conductive states with exceptional tunability and stability. Since interface RS does not require the formation of a continuous Ag filament via Ag^+^ ion reduction, it can achieve an ultralow switching energy of ∼0.2 fJ. Furthermore, a hardware-based image processing with a software-comparable computing accuracy is demonstrated using the flexible Ag_2_S memristor array. And the image processing with interface RS indeed consumes 2 orders of magnitude lower power than that with filamentary RS on the same hardware. This study demonstrates a new resistance–switching mechanism for energy-efficient flexible neural network hardware.

## 1 Introduction

Multiply accumulate calculations (MACs) are a core algorithmic operation of digital matrix processing and play an essential role in modern information technology.^1,2^ They are capable of extracting specific features from original data to achieve further analysis and computation, which has revolutionized big data processing technologies in human activities.^3^ In past decades, the hardware implementation that supports MAC operation has been built with modern computers constructed by von Neumann architecture, in which the separated data processing and storage units consume most of the energy and time in data transfer.^4^ The resulting large power dissipation and significant data latency would inevitably increase the chip temperature and degrade computing performance.^5^ Recent progress in memristor technology provides a promising solution to address this problem. Memristor is capable of changing its resistance under external electric field. This resistance–switching (RS) behavior enables data storage via device conductance modulation.^6,7^ Moreover, the memristor array can perform MAC operation directly using Ohm’s law and Kirchhoff’s current law, realizing parallel data storage and processing within a single unit.^8,9^ It can therefore avoid the extensive data shuttling during multistep multiplications and additions, thus significantly reducing the energy consumption and data latency.

Highlighting the versatility of a memristor-based computing system, memristor-based wearable electronics toward smart applications,^10,11^ e.g., electronic skin, artificial perception, and health monitoring, have recently attracted significant research attention. Since these flexible electronics are normally powered by batteries, real-time data processing with exceptional energy efficiency are greatly desired to extend the endurance of the power supply. This demands both flexibility and low power consumption of the memristor devices in the wearable electronics.^12^ However, current flexible memristors (FMs) mostly switch on the formation and ablation of conductive filaments, which puts large energy demands at setting/resetting processes, even though nanometer-thick electrolyte films are utilized.^15−17^ In addition, working states formed with highly conductive filaments access ultralow resistances, which results in high currents and thus consumes large amounts of power in the computing process.^18^ Although recent advances show the improved energy efficiency of FM to some extent, the RS mechanism is still filament-based. FMs with low switching energy at ∼fJ level, which is comparable to that of biological synapses, are rarely reported. Furthermore, the advances of FMs for neuromorphic computing are mostly demonstrated in software-based simulations instead of real FM array hardware,^12,19,20^ taking idealized single FM device performance (without considering conductance drift, device-to-device variation, etc.) as the input.

Recent progress on intrinsically flexible inorganic semiconductors offers a tantalizing opportunity to address the aforementioned problems. Ag_2_S is an n-type semiconductor, with extraordinary ductility at room temperature.^21,22^ In our previous work, we demonstrate an Ag_2_S-based full-inorganic flexible memristor that exhibits a record high 10^6^ ON/OFF ratio.^23^ This exceptional ON/OFF ratio is induced by sequential processes of Schottky barrier height (SBH) modification at the contact interface and filament formation inside the electrolyte. High-voltage pulses (over the threshold voltage ∼ 0.4 V) set the memristor into the 10^–4^ to 10^–2^ S range by forming/ablating Ag filaments (noted as filament-type memristor, FTM), while low-voltage pulses drive the device to a relatively lower conductance range (about 10^–6^ to 10^–4^ S) by modifying the SBH of the contact interface (noted as interface-type memristor, ITM). In this work, we demonstrate that RS can be achieved solely with SBH modification at the Ag/Ag_2_S interface. The unique interface RS can be facilitated by a smaller electrical bias (±0.2 V) and exhibits exceptional switching endurance (10^5^ switching cycles) and retention (10^4^ s). Moreover, a significantly reduced switching energy (∼0.2 fJ) is achieved with the interface RS, which is several orders of magnitude smaller than the reported filamentary FMs. MAC operation on an Ag_2_S FM array is also implemented to demonstrate a hardware-based image processing task, where 2 orders of magnitude lower power consumption is achieved with interface RS than that with filamentary RS on the same device array.

## 2 Results and Discussion

### 2.1 Bipolar Interface RS under Small Bias

**Figure 1:**
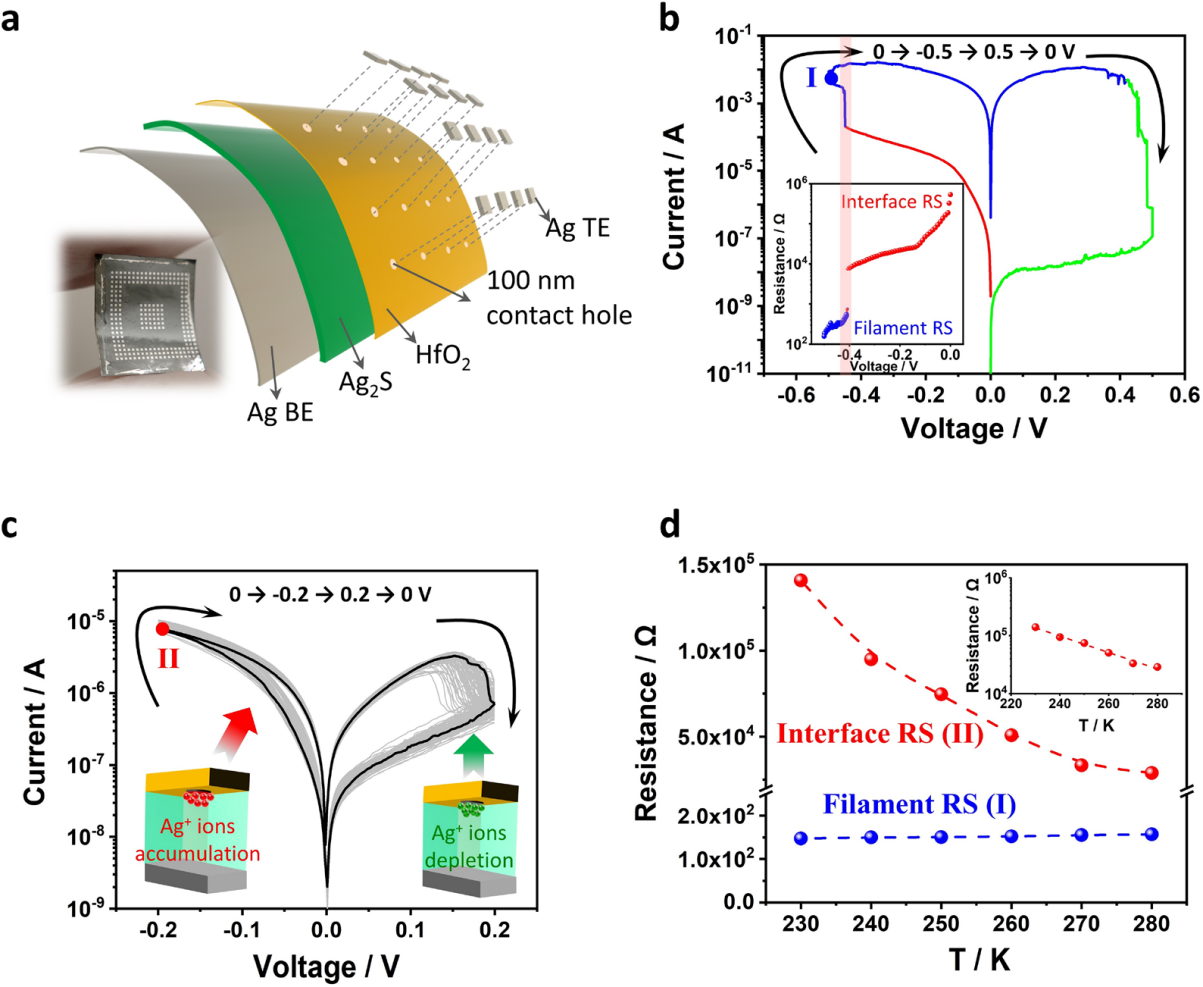
Full-inorganic Ag_2_S flexible memristor. (a) Schematic illustration of FM structure. The device consists of a silver top electrode (Ag TE), 5 nm thick HfO_2_ dielectric layer (through which 100 nm contact holes are etched), 100 μm thick Ag_2_S electrolyte, and silver bottom electrode (Ag BE). The photography in the lower left shows the bended Ag_2_S FMs. (b) Current (*I*)–voltage (*V*) characteristics of FM under 0 V → −0.5 V → 0.5 V → 0 V voltages applied to the Ag TE.^23^ The inset shows resistance reduction under negative setting bias, where interface RS (setting bias < −0.4 V) is observed before filament formation. Device set by −0.5 V bias is marked by stage I in the curve. (c) *I*–*V* characteristics of FM under 0 V → −0.2 V → 0.2 V → 0 V voltages applied to the Ag TE. As schematically illustrated, the device is set by the Ag^+^ ion accumulation induced SBH reduction and reset by the Ag^+^ ion depletion induced SBH increase at the top contact interface. Device set by −0.2 V bias is marked by stage II in the curve. (d) Device resistance evolution under temperature variations. Different resistance–temperature dependences indicate filament RS for stage I and interface RS for stage II. The inset shows an exponential relationship between device resistance (at stage II) and temperature.

As depicted in Figure 1a, the Ag_2_S-based FM comprises a bottom silver electrode and a free-standing Ag_2_S film as both functional electrolyte and flexible substrate interfaced with a top silver electrode via a 100 nm contact hole formed in a 5 nm thick HfO_2_ electron barrier layer.^23^ A bipolar RS with an ON/OFF ratio close to 10^6^ can be achieved using −0.5 V/0.5 V set/reset biases, as reported in our previous work^23^ (also see Figure 1b). This high ON/OFF ratio is induced by sequential processes of Schottky barrier modification at the contact interface (set bias < −0.4 V) and nanoscale Ag filaments formation inside the electrolyte (set bias > −0.4 V), with an abrupt resistance reduction between them (see the inset of Figure 1b). The Schottky barrier modification only requires Ag^+^ ion migration, while continuous Ag filament formation inside the electrolyte requires additional electrochemical reduction of the migrated Ag^+^ ions at the cathode.^24,25^ The RS based on Ag filaments is therefore more energy-demanding. In addition, the formed nanoscale metallic filaments are relatively unstable, which generate challenges in analogue conductance tunability.^20,23^ Herein we demonstrate the RS based only on the interface Schottky barrier modification in our Ag_2_S FMs with reduced setting/resetting voltages for energy-efficient computing applications. As shown in Figure 1c, reversible RS is achieved under 0 V→ −0.2 V → 0.2 V → 0 V bias, where the setting/resetting processes are induced only by SBH reduction/increase at the top interface. Noticeably, no abrupt current increase is observed in the set process, indicating no filament formation in the Ag_2_S electrolyte. To further confirm this, we employed in situ cryogenic measurement to record the change of device resistance (after setting) under temperature variation. As shown in Figure 1d, the device after −0.2 V setting voltage exhibits an exponential resistance–temperature relationship (stage II in Figure 1d and its inset), indicating a typical thermal emission process in the carrier transportation.^26^ This carrier transportation behavior confirms that device resistance is still dominated by the Schottky junction at Ag/Ag_2_S interface after −0.2 V setting.^23^ In direct contrast, the device resistance after setting with −0.5 V voltage (stage I, after the Ag filament formation) shows weak linear dependence on the temperature, which is the characteristic behavior of phonon scattering effect in metallic conductors.^27−29^

### 2.2 Endurance and Retention of Interface RS

**Figure 2:**
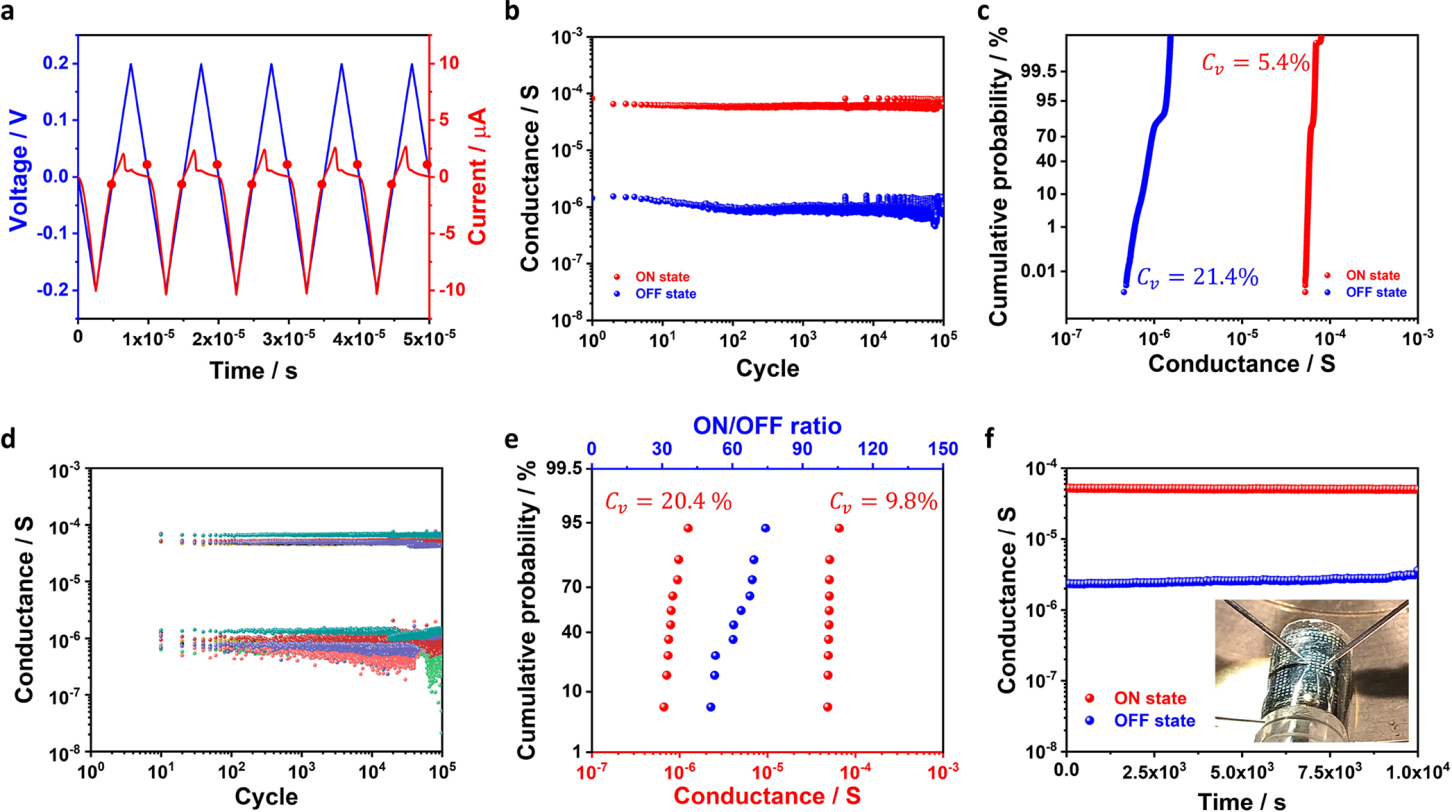
Endurance and retention of interface RS. (a) Recorded current (with high temporal resolution) under pulsed voltage stresses of 5 cycles. The red points illustrate the two data points recorded in each cycle in the following low temporal resolution measurements. (b) ON/OFF states conductance of a Ag_2_S-based memristor recorded for 10^5^ repeating cycles. The reading voltage is ±5 mV as marked in panel a, and the data are collected for each switching cycle. (c) Cumulative probability of ON/OFF states conductance in 10^5^ endurance cycles. (d) ON/OFF states conductance of 10 Ag_2_S-based memristors. The conductance is collected every 10 switching cycles. (e) Cumulative probability of the averaged ON/OFF states conductance and switching ratio of the 10 devices. The ON/OFF states conductance of each device is averaged from 10^5^ endurance cycles. (f) Data retention of ON and OFF conductance recorded under bending condition (with 3 mm curvature radius).

To investigate the endurance of interface RS, a sequence of triangular ±0.2 V pulsed voltage was applied to the device and the current was simultaneously recorded. As reflected by the current trance in Figure 2a, the Ag_2_S device exhibits repetitive responses to setting/resetting biases. To conduct further endurance testing, current measurement with a low temporal resolution was employed for 10^5^ switching cycles. In each cycle, only 2 data points representing the ON/OFF states conductance after setting/resetting processes (as illustrated by the red points in Figure 2a) were recorded. As summarized in Figure 2b,c, interface RS could be stably operated over 10^5^ cycles, with ON/OFF states conductance clustered between (7 ± 2) × 10^–5^ and (8 ± 3) × 10^–7^ S, respectively. The coefficient of variations (*C*_v_, calculated as the standard deviation divided by the mean value) of ON/OFF states conductance are 5.4 and 21.4%, demonstrating a small cycle-to-cycle variation. To evaluate the device-to-device variation, we conduct the endurance measurement for 10 Ag_2_S memristors, with ON/OFF states recorded every 10 cycles for each device (Figure 2d). We averaged the conductance over 10^5^ switching cycles of each device, and further calculated the cumulative probability of switching ratio. The averaged ON/OFF states conductance of 10 devices exhibit the coefficient of variations at 9.8 and 20.4%, with an ON/OFF ratio range between 50 and 70 (Figure 2e).

The stability of device performance under bending condition is important for flexible applications. In this work, 10 Ag_2_S memristors were bent with a curvature radius of 3 mm and then recovered to a flat state for electrical measurements. The ON/OFF states conductance evolution against 1000 bending cycles is summarized in the Supporting Information (SI) Figure S1, where a reproducible interface RS behavior under bending condition is shown. Moreover, we further test the retention when the Ag_2_S device is kept bent (with 3 mm bending radius as shown in the inset of Figure 2f). Compared with some other interface-type memristors (as summarized in SI Table S1), the long-term stability over 10^4^ s demonstrates significantly improved data retention under a much smaller switching voltage. The result indicates that Ag^+^ ion migration can modulate SBH more efficiently than the reported methods (e.g., trapping/detrapping of charged carriers in interface states or the field-induced oxygen vacancy migration).

### 2.3 Tunability, Stability, and Switching Energy of Interface RS

**Figure 3:**
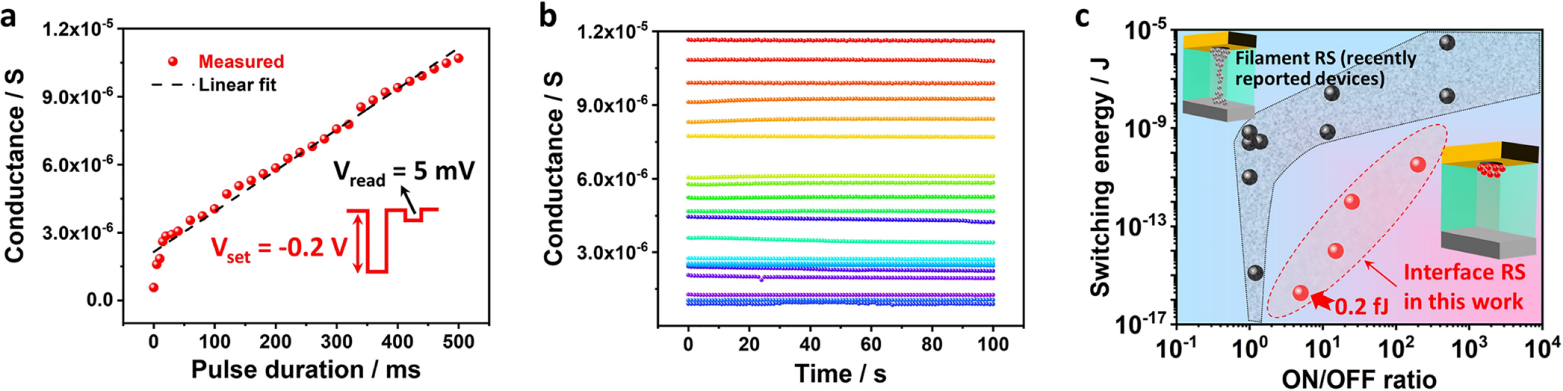
Tunability, stability, and switching energy of interface RS. (a) Conductance evolution of Ag_2_S device under −0.2 V setting pulse. The conductance shows a linear dependence on the pulse duration, which demonstrates the exceptional tunability of multiple-level interface RS states. (b) Retention of 20 interface RS conductive states. Further retention of 8 randomly selected intermediate memory states can be found in SI Figure S2. (c) Comparison of switching energy between Ag_2_S device (with interface RS) and recently reported filamentary devices. A significant reduction of switching energy is demonstrated with interface RS.

Multiple-level conductive states are required for synaptic weight update in a memristor-based artificial neural network (MANN) and therefore play an essential role in computing tasks.^30,31^ Since postsynaptic current in MANN scales directly with the conductance of the state, low-conductance states hold great promise for energy-efficient computing. With this understanding, we further investigate the tunability, stability, and switching energy of interface RS states. The efficient modulation of low-conductance states in ITM can be realized by synaptic duration dependence plasticity (SDDP), as demonstrated by applying −0.2 V pulses with variable durations (Figure 3a). The conductance change exhibits a linear dependence to the pulse duration, with time scale down to the milliseconds level. Besides, the nonvolatility of 20 multiple-level states is further verified by the retention measurements shown in Figure 3b. The tunability and stability of multiple conductive states promise the programming reliability of interface RS for the synaptic weight update in MANN. More importantly, the switching energy of interface RS in our device is indeed much smaller than the reported FM based on filament RS. As summarized in Figure 3c, the pulse energy required to trigger interface RS with different ON/OFF ratios is benchmarked with recently reported filamentary FMs,^15,16,32−36^ where a reduction of switching energy by several orders of magnitude is achieved. An ultralow switching energy of only ∼0.2 fJ is needed to achieve a 5 ON/OFF ratio with the interface RS in our Ag_2_S FM (see the calculation details of switching energy in SI Figure S3). This demonstrates a very promising strategy to reduce memristor power dissipation with this new RS mechanism.

### 2.4 Hardware-Based Image Processing Task on Ag_2_S Device Array

**Figure 4:**
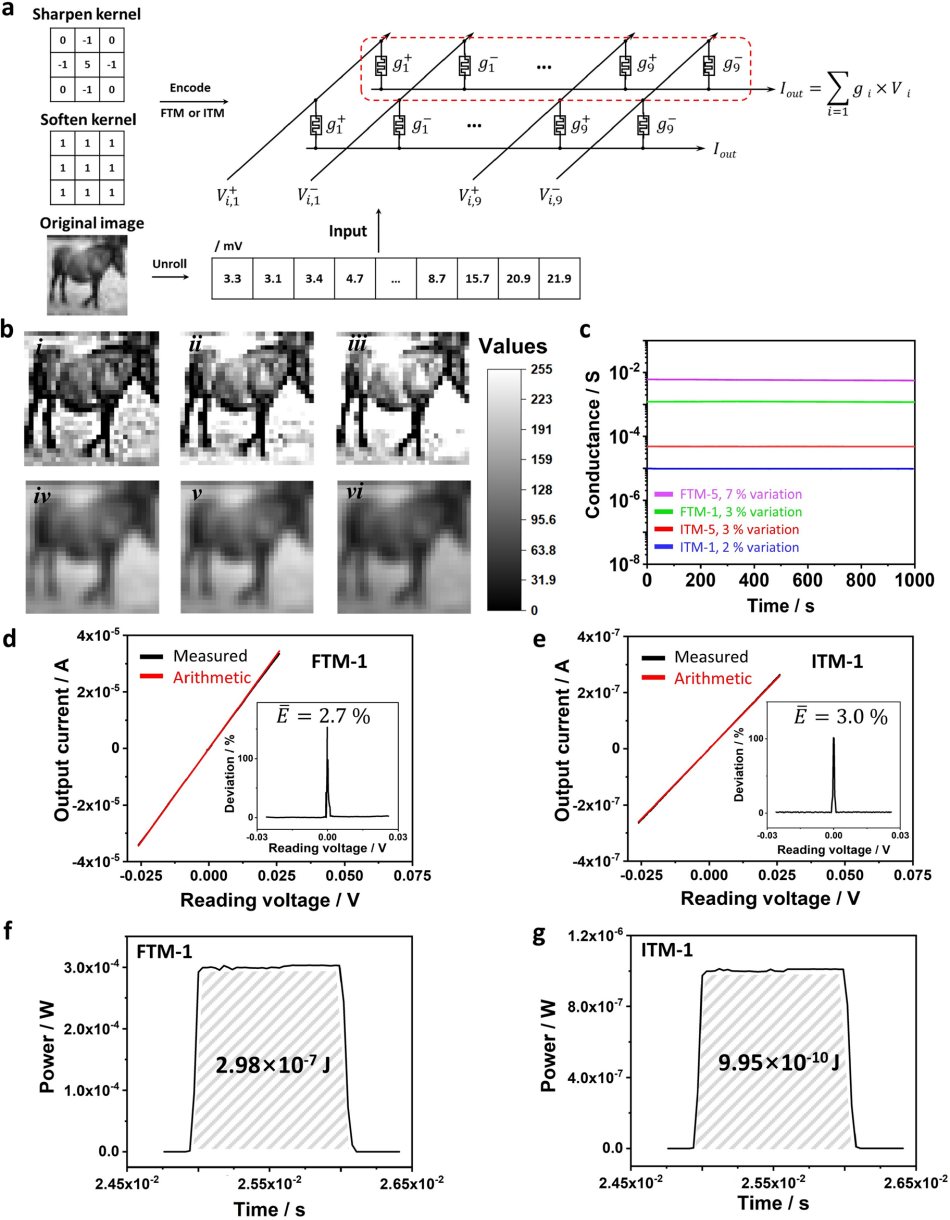
Image processing demonstration using Ag_2_S FM array. (a) Convolutional kernel values are encoded into either an FTM or ITM Ag_2_S device array. The grayscale value of each pixel of the original image is mapped to the input voltage, which is fed to the top electrodes of different devices via a 3 × 3 input block. The postsynaptic current after MAC operation is recorded at the bottom electrode and back-transferred to grayscale values to generate the output image. (b) Software-based simulation result (i) and hardware outputs of FTM (ii) and ITM (iii) after sharpening operation. Software-based simulation result (iv) and hardware outputs of FTM (v) and ITM (vi) after softening operation. (c) Retention measurement of conductive states used in the image processing. (d) Measured (from the hardware) and arithmetic current (from the simulation) of FTM-1 under input reading voltages. The inset shows the current deviation distribution and its average value. Since the output current around ±0.4 mV is close to the background noise level, a significant deviation is generated when applying small reading voltages. This only contributes to the error for grayscale values below 4 in our demonstration. (e) Measured (from the hardware) and arithmetic current (from the simulation) of ITM-1 under input reading voltages. The inset shows the current deviation distribution and its average value. (f) Pulse power against elapsed time in the kernel encoding process for FTM-1. The energy consumption is calculated by integrating the pulse power against the device setting time. (g) Pulse power against elapsed time in kernel encoding process for ITM-1.

To further study the practical computing task using our Ag_2_S memristor-based hardware, we perform MAC operations on a single-dot device array (which can be logically treated as a 1 × *N* cross-bar structure) to demonstrate a hardware-based image processing task. As shown in Figure 4a, the “sharpen” and “soften” convolutional kernel values are mapped to the FM conductance in an Ag_2_S device array. For comparison, the kernel values are coded to two FM arrays with filament or interface RS, respectively (containing FTM-1, FTM-5, ITM-1, and ITM-5 encoded with kernel values 1 and 5; see SI Figure S4 for kernel encoding details). The pixel values (ranging from 0 to 255) of the original image were linearly mapped into reading voltages (ranging from 0 to 25.5 mV in amplitude; see SI Figure S5). Since the device array shares one bottom electrode, the output current generated by the voltage–conductance multiplication and the current addition can be collected after applying read voltages to the top electrodes of the 18 FMs in the array (the operation details can be found in Methods). This output current represents the convoluted feature map and can be decoded to the output grayscale image for visualization. Figure 4b shows the decoded output images from both software simulation (i and iv) and hardware processing (ii, iii, v, and vi). The simulation results are obtained from the accurately designed kernels and, thus, can be utilized as the reference to evaluate the processing performance of hardware.^31^ In FTM- and ITM-based outputs, the contrast between the “horse” and its surroundings is significantly enhanced after the sharpening operation, while the softening operation smooths the item with its surrounding pixels. The comparable experimental and simulation results demonstrate the potential of the Ag_2_S device for artificial neural network hardware.

The hardware-processed results could be slightly different from the software results, due to the conductance drift of memristor devices. In software-based results, the kernel value is fluctuation-free but such variation is unavoidable in hardware processing. We recorded the variation of conductive states utilized for sharpen kernel encoding, where slight conductance decay is observed for both FTM and ITM (Figure 4c). Moreover, the conductance variation against the input voltage can also affect the hardware results. As depicted by Figure 4d,e, the deviation between output current and arithmetic current (from simulation) exists across the reading voltage window, with the average values of 2.7% and 3.0% for FTM-1 and ITM-1, respectively. The slightly larger deviation is also observed in ITM-5 (compared with FTM-5; see SI Figure S6), which can be attributed to the fact that the top Schottky contact resistance in the ITM device is relatively more sensitive to the reading voltages than the silver filament in FTM. The small input reading voltage can induce slight Ag^+^ ion migration and modify the SBH in ITM, whereas the dissolution of the silver filament in FTM needs extra energy in Ag atom oxidation.

Interface RS indeed exhibits improved energy efficiency compared to that of FTM in hardware-based computing. In this image processing demonstration, the total energy consumption is contributed from the convolutional operation and the kernel encoding processes. For convolutional operation, the multiplication and addition are naturally performed after applying reading voltages, during which the power density is directly scaled with the device conductance. The ITM array (with ∼10^–5^ S conductance) could thus reduce the energy consumption by 2 orders of magnitude compared to FTM (with ∼10^–3^ S conductance). We further calculate the power consumption of the kernel encoding process, by integrating the power of the setting pulse against the device setting time. The kernel encoding in ITM-1 consumes 9.95 × 10^–10^ J, which is about 300 times smaller than that in FTM-1 (Figure 4f,g). The greatly reduced power consumption, with simulation-comparable processing accuracy, demonstrates the benefit of utilizing low-conductance interface RS for energy-efficient computation at the hardware level. Finally, we note that the dot–point device array used in this work serves for the proof-of-concept computing demonstration based on interface RS-based flexible memristor hardware. The physical cross-bar flexible device array, with the engineering issues in integrated circuit design (e.g., the line resistance and sneak paths) considered, will be investigated in our future work.

## 3 Conclusion

We demonstrate a unique interface RS in full inorganic flexible Ag_2_S memristor, with no need of filament formation/ablation in the solid electrolyte. The interface RS can achieve a much smaller switching energy of ∼0.2 fJ, compared to conventional FM with filamentary RS. Moreover, real hardware-based image processing tasks are performed on our Ag_2_S FM array. Image processing based on interface RS indeed shows 2 orders of magnitude lower energy consumption than using filament RS on the same FM array. This study provides a novel promising RS mechanism toward energy-efficient neural network hardware.

## 4 Methods

### 4.1 Ag_2_S-Based Memristor Fabrication and Characterization

The Ag_2_S film with 100 μm thickness was synthesized from the solid-state element reaction. The details of device fabrication and characterization can be referenced in our previous work.^23^

### 4.2 Hardware-Based Image Processing Task

Sharpening and softening kernels were encoded into memristors for image processing demonstration. Each kernel had 3 × 3 pixels, and two memristors were used to represent the positive and negative weight values for each kernel pixel. Specifically, the multiplication and addition through a negative kernel value “–1” were performed by collecting the net current through a high resistance state device (with a positive input voltage, the subcurrent is negligible) and a low resistance state device (with a negative input voltage, the subcurrent is dominating). An image with 32 × 32 original pixels was split into 3 × 3 input matrixes and then transformed to input presynaptic reading voltages, which were continuously fed to the top electrodes of the kernel memristors. Since these devices share one common large bottom electrode, the collected postsynaptic current is multiplicated by Ohm’s law and accumulated by Kirchhoff’s current law. The measured postsynaptic current was contributed from the 3 × 3 input pixel matrix in the original image, which corresponds to a typical convolutional operation in software-based simulation. After feeding 16200 reading pulses (30 × 30 × 18, without padding process) to kernel memristors, all of the pixel information can be collected and the convoluted image can be decoded.

## Supporting Information Available

ON/OFF states conductance of 10 devices, 5000 s retention of randomly selected intermediate memory states, switching energy calculation, convolution kernel encoding with device conductance, transition of grayscale values to input reading voltages, conductance drifts, and comparison of interface-type memristors based on Schottky barrier height modification (PDF)
