# Supplementary material for: Full-Inorganic Flexible Ag2S Memristor with Interface Resistance–Switching for Energy-Efficient Computing
Source: ACS Appl Mater Interfaces. 2022 Sep 14;14(38):43482–9. doi: 10.1021/acsami.2c11183 (PMC9523614; doi:10.1021/acsami.2c11183)
Supplement: Supplementary file 1 — am2c11183_si_001.pdf [file am2c11183_si_001.pdf]

Supporting Information for

A full-inorganic flexible Ag_2S memristor with interface resistance-switching for energy-efficient computing

Yuan Zhu[†], *Jia-sheng Liang*[‡], *Xun Shi*[‡,*] & *Zhen Zhang*[†,*]

[†]Division of Solid-State Electronics, Department of Electrical Engineering, Uppsala University, Uppsala 75121, Sweden.

[‡]State Key Laboratory of High Performance Ceramics and Superfine Microstructure, Shanghai Institute of Ceramics, Chinese Academy of Sciences, Shanghai 200050, China.

*Corresponding author. Email: zhen.zhang@angstrom.uu.se (Z.Z.); xshi@mail.sic.ac.cn (X.S.).

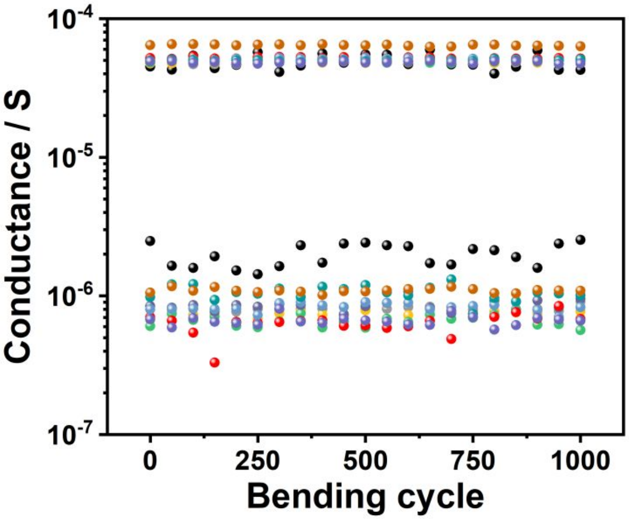

Figure S1. The ON/OFF states conductance of 10 devices under bending test. The device was first bent with 3 mm curvature radius and then got recovered to flat state for the electrical measurements.

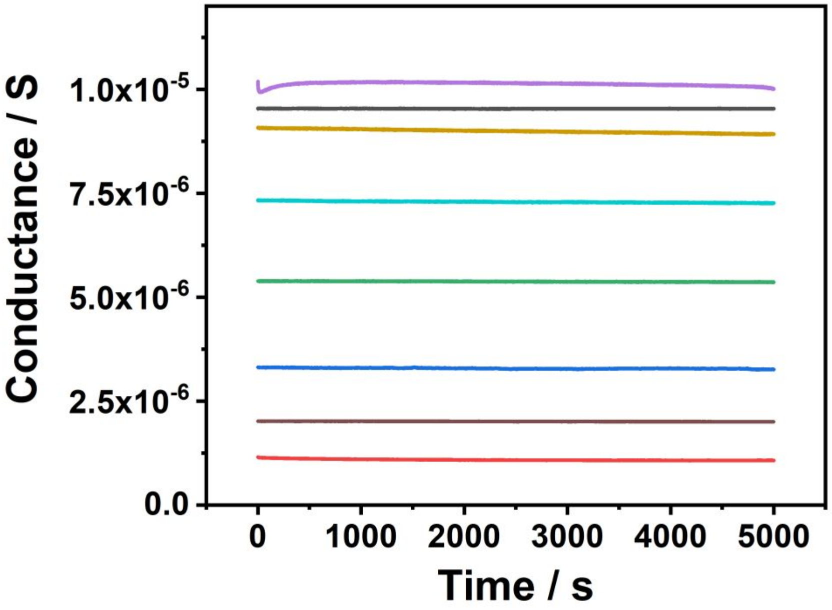

Figure S2. The 5000 s retention of 8 randomly selected intermediate memory states.

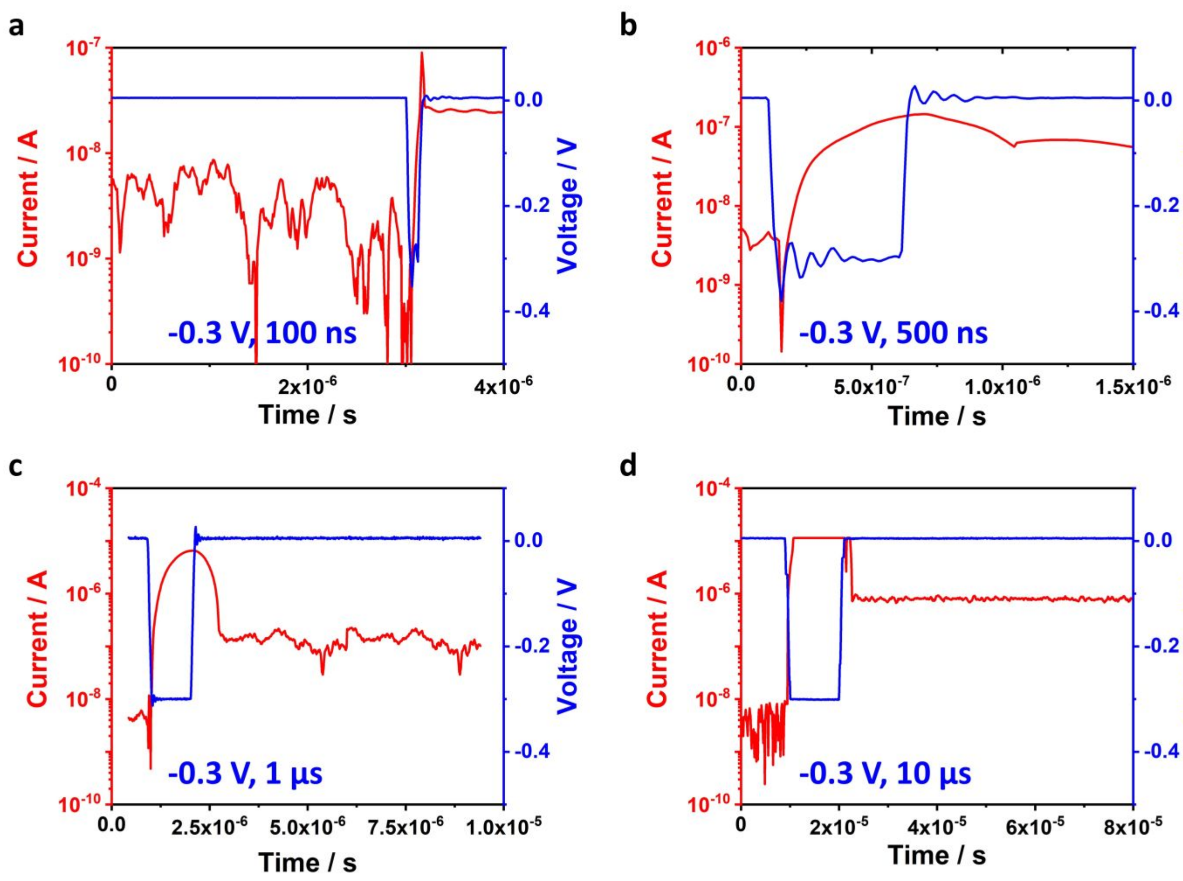

Figure S3. Switching energy of an Ag_2S memristor with interface RS. The device current and applied voltage are recorded before and after pulse: (a) -0.3 V, 100 ns pulse; (b) -0.3 V, 600 ns pulse; (c) -0.3 V, 1 μs pulse; (d) -0.3 V, 10 μs pulse. The switching energy is calculated by integrating current, voltage during pulse duration.

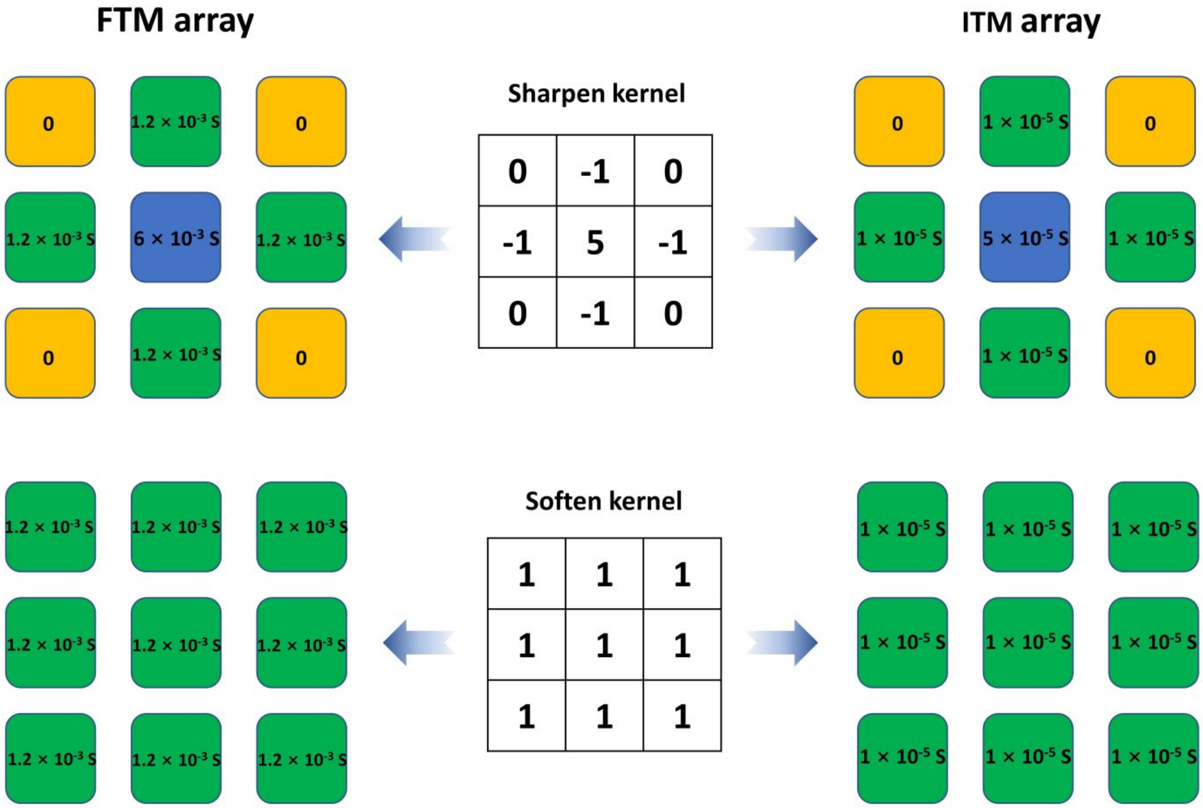

Figure S4. The convolution kernel encoding process. The kernel values were linearly mapped to the conductance and encoded into both FTM and ITM in LTP mode. Please note that the kernel values "-1" and "1" are encoded into the same conductance level, with the convolution operated with different input voltage polarity (the details can be found in Method in manuscript).

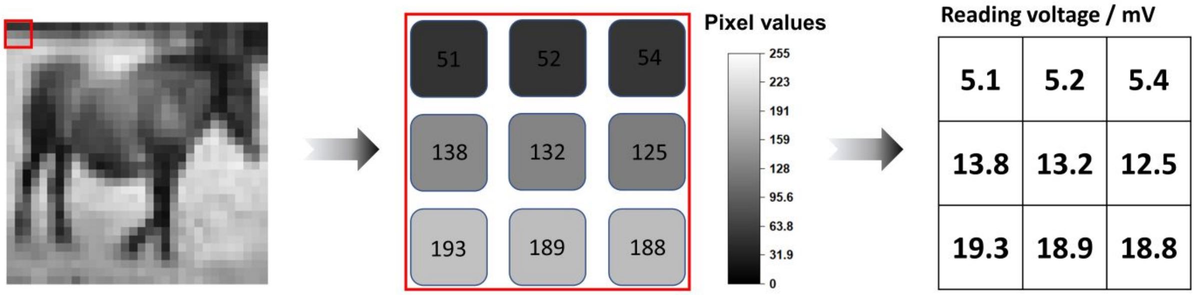

Figure S5. The transition of greyscale values to input reading voltages. The greyscale values ranging from 0 to 255, were linearly mapped into reading voltages (0 mV to 25.5 mV). A 3×3 input block was utilized to perform convolution operation and generate one output pixel value. After sliding the input block along with the entire original image, an output image can be obtained.

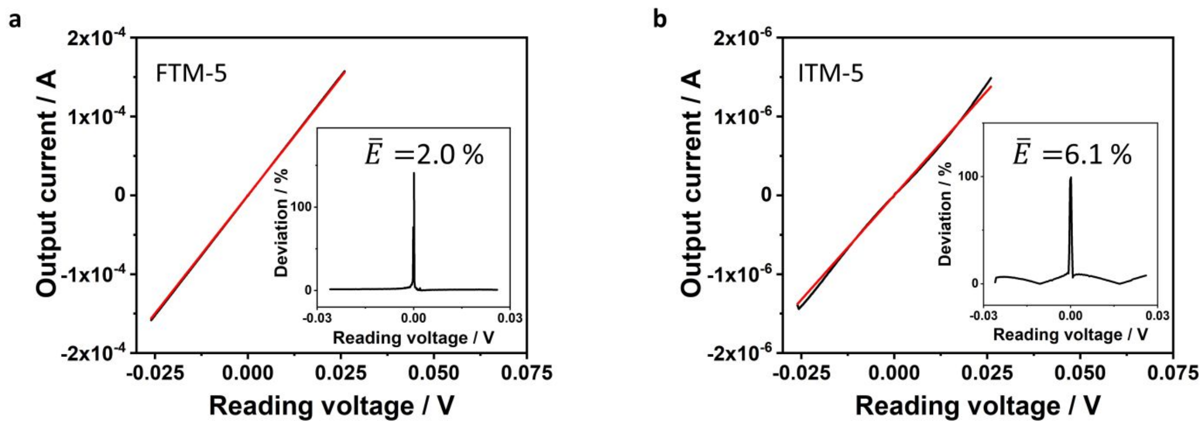

Figure S6. The conductance drift against input reading voltages. (a) The measured (from the hardware) and arithmetic current (from the simulation) of FTM-5 under input reading voltages. The inset shows the current deviation distribution and its average value. (b) The measured (from the hardware) and arithmetic current (from the simulation) of ITM-5 under input reading voltages. The inset shows the current deviation distribution and its average value.

Table S1. Comparison of interface-type memristors based on Schottky barrier height modification

Electrolyte	Switching mechanism	Set voltage/V	ON/OFF ratio	Retention/s	Endurance	Application	Ref.
Nb:$SrTiO_3$	trapping/detrapping carriers	5	100	/	/	/	1
$SrTiO_3$	trapping/detrapping carriers	2.5	10000	/	/	/	2
CoO_x	trapping/detrapping carriers	5	5	< 0.1	1000	/	3
TiO_x	Oxygen migration	1.5	10	1×10^4	100	/	4
Ag_2S	Silver migration	0.2	50-70	1×10^4	1×10^5	Image processing	This work

Reference

(1) Li, G.; Yang, Z.; Zhang, X.; Li, G.; Sun, X.; Zhang, W. Origin of Resistance Switching and Regulation of the Resistance Stability by the Interface State Density on the Pt/Nb:SrTiO3 Interface. *Phys. Status Solidi A* **2019**, *216* (18), 1900125.

(2) Sun, X.; Li, G.; Chen, L.; Shi, Z.; Zhang, W. Bipolar Resistance Switching Characteristics with Opposite Polarity of Au/SrTiO3/Ti Memory Cells. *Nanoscale Res. Lett.* **2011**, *6* (1), 599.

(3) Fu, J.; Hua, M.; Ding, S.; Chen, X.; Wu, R.; Liu, S.; Han, J.; Wang, C.; Du, H.; Yang, Y.; Yang, J. Stability and Its Mechanism in Ag/CoOx/Ag Interface-Type Resistive Switching Device. *Sci. Rep.* **2016**, *6* (1), 35630.

(4) Park, J.; Biju, K. P.; Jung, S.; Lee, W.; Lee, J.; Kim, S.; Park, S.; Shin, J.; Hwang, H. Multibit Operation of TiOx -Based ReRAM by Schottky Barrier Height Engineering. *IEEE Electron Device Lett.* **2011**, *32* (4), 476–478.
